# New tracheal stainless steel stent pilot study: twelve month follow-up in a rabbit model

**DOI:** 10.7717/peerj.7797

**Published:** 2019-10-08

**Authors:** Sandra Lopez-Minguez, Carolina Serrano-Casorran, Jose A. Guirola, Sergio Rodriguez-Zapater, Cristina Bonastre, Miguel Angel De Gregorio

**Affiliations:** 1 University of Zaragoza, GITMI Minimally Invasive Techniques Research Group, Zaragoza, Spain; 2 Animal Pathology, University of Zaragoza, Zaragoza, Spain; 3 Pediatrics, Radiology and Phisical medicine, University of Zaragoza, Zaragoza, España; 4 Interventional Radiology Service, Lozano Blesa University Hospital Zaragoza, Spain

**Keywords:** Tracheal collapse, Stent, Tracheomalacia, Dogs, Pilot study, Interventional radiology, Minimally invasive surgical procedures, Device removal, Airway management, Respiratory pathology

## Abstract

**Background:**

Canine tracheal collapse is a complex airway pathology without promising treatment results. Currently nitinol stents are the best surgical option; however, some professionals are doubting if stent placement is the best option due to the associated complications.

**Objective:**

Determine the technical feasibility, safety, and long-term follow-up after the implantation of a new tracheal stent designed for canine tracheal collapse.

**Methods:**

Thirteen healthy, adult female New Zealander rabbits were involved in this pilot study**.**A new intra-tracheal device (Reference number 902711 patent registered as CasMin-Twine) was implanted in ten animals. Deployment was performed under general anesthesia, making a puncture incision via a 21 Gauge needle in the intra-tracheal space where the stent was introduced with a screwing process. The device was fixed to the tracheal wall with a non-absorbable suture. Computerized Tomography (CT) and an endoscopy to study structural abnormalities were performed after 30, 90 and 365 days after stent placement.

**Results:**

Technical and clinical success was 100%. There was no significant change in behavior or respiratory disorders. CT studies showed no significant alterations. After the 30 days, 60% of the animals showed partial endothelization in the endoscopy study, and only one animal still presented partial endothelization after 12 months. Mucus accumulation was only present in 40% of cases and classified as low, without respiratory consequences. Only one animal presented a single granuloma at caudal stent tip.

**Conclusions:**

This new tracheal stent (CasMin-Twine) is an effective and safe procedure with promising results, and also shows the possibility of removing the device after endothelization has been produced. New studies should be carried out to evaluate the effectiveness in patients with tracheomalacia.

**Clinical Significance/Impact:**

This new product can give veterinarians a new option of treatment for this complicated pathology. Minimizing specific equipment for its deployment, CasMin-Twine will be more accessible for all professionals.

## Introduction

Canine tracheal collapse is a progressive, degenerative disease occurring mainly in middle-aged small and toy breeds with a high incidence in small dogs and prevalence reported in 1974 about 0.5% of 2,780 dogs (Amis, 1974).

The etiology is not well understood, but it has been observed that a degeneration of the tracheal cartilage rings as a result of hypo-cellularity and decreased glycosaminoglycan content (Amis, 1974) that leads to cartilage weakness and loos of the C shape morphology and laxity of the dorsal tracheal membrane can be altered, resulting in a dynamic collapse during respiration. The disease can be focal or generalized and in many cases associated with bronchial degeneration (Amis, 1974; Tappin, 2016; Sura & Krahwinkel, 2008; Beranek, Jaresova & Rytz, 2014).

The clinical manifestations depend on the severity of the collapse and the affected segment of the tracheal, which can progressively degenerate in time. Clinical symptoms can range from an exercise intolerance with a classic “goose-honk” to severe respiratory distress and syncope (Tappin, 2016; Sura & Krahwinkel, 2008; Beranek, Jaresova & Rytz, 2014; Weisse et al., 2008; Chisnell & Pardo, 2015). The diagnostic procedure is usually performed by fluoroscopy and endoscopy. However, dynamic CT can play a major role in the diagnosis of this pathology (Ullmann et al., 2018).

Medical management is the first option, being palliative and temporally (Tappin, 2016; Ullmann et al., 2018). Many dogs improve with anti-inflammatory drugs, antitussives, antibiotics, bronchodilators and weight-loss, but in high grade collapse it is usually limited.

If options for medical therapy have failed to control symptomatology, surgery should be considered. Surgical options are also palliative, they can be varied and different according to the collapsed area. Extra-luminal C-shaped plastic rings can be only used in non-intra-thoracic collapse; it is an invasive surgery with associated complications. In a previous study (Chisnell & Pardo, 2015), the technical success was 65%, with 17% of the dogs presented laryngeal paralysis and 9% needed a second intervention. Moreover, high morbidity rates are associated with surgical reconstruction of thoracic tracheal segments (Chisnell & Pardo, 2015; Tinga et al., 2015).

Because of these complications and the improvement of new surgical methods, various minimally invasive techniques have been investigated for the deployment of intraluminal devices. Currently, the use of self-expandable nitinol stents is recommended due to the complications arising of stainless-steel stents, which include migration, shortening and fracture. Self-expandable nitinol stents presented better results and are the treatment of choice when medical management has failed; however, granuloma formation and stent migration are also described as complications (Sura & Krahwinkel, 2008; Beranek, Jaresova & Rytz, 2014; Weisse et al., 2008; Chisnell & Pardo, 2015; Ullmann et al., 2018; Clarke, 2018; Tinga et al., 2015; Rosenheck et al., 2017; Serrano et al., 2016; Moritz, Schneider & Bauer, 2004; Chung et al., 2011).

The search for a better intra-tracheal device is required, and long-term follow-up studies will be necessary. In this study, we proposed an original approach for tracheal collapse, and a new intra-tracheal device assessed in laboratory animals with a 12-month follow-up. The main purpose of this research is to evaluate the new tracheal approach and long-term complications arising from this new surgical technique and the safety and efficacy of the device.

## Material and Methods

This study was approved by the ethics committee PI26/17- University of Zaragoza, Spain. This study was elaborated according to ARRIVE guidelines (Kilkenny et al., 2010).

### Laboratory animals

A total of thirteen healthy New Zealand adult rabbits was involved in this pilot study. The follow-up was scheduled for 12 months to analyze long-term complications. Animals were obtained from the experimental center of our institution (University of Zaragoza), with weights ranging between 4.2–4.5 kg. Three control rabbits (3/13) were compared in this study. All veterinarians and personnel in charge of the animals had the required qualifications, RD 1021/2005. The experimental animal was elected based on characteristics already described in previous studies (Serrano et al., 2016).

### Experimental procedure (Pilot study)

#### Experimental device

**Figure 1:**
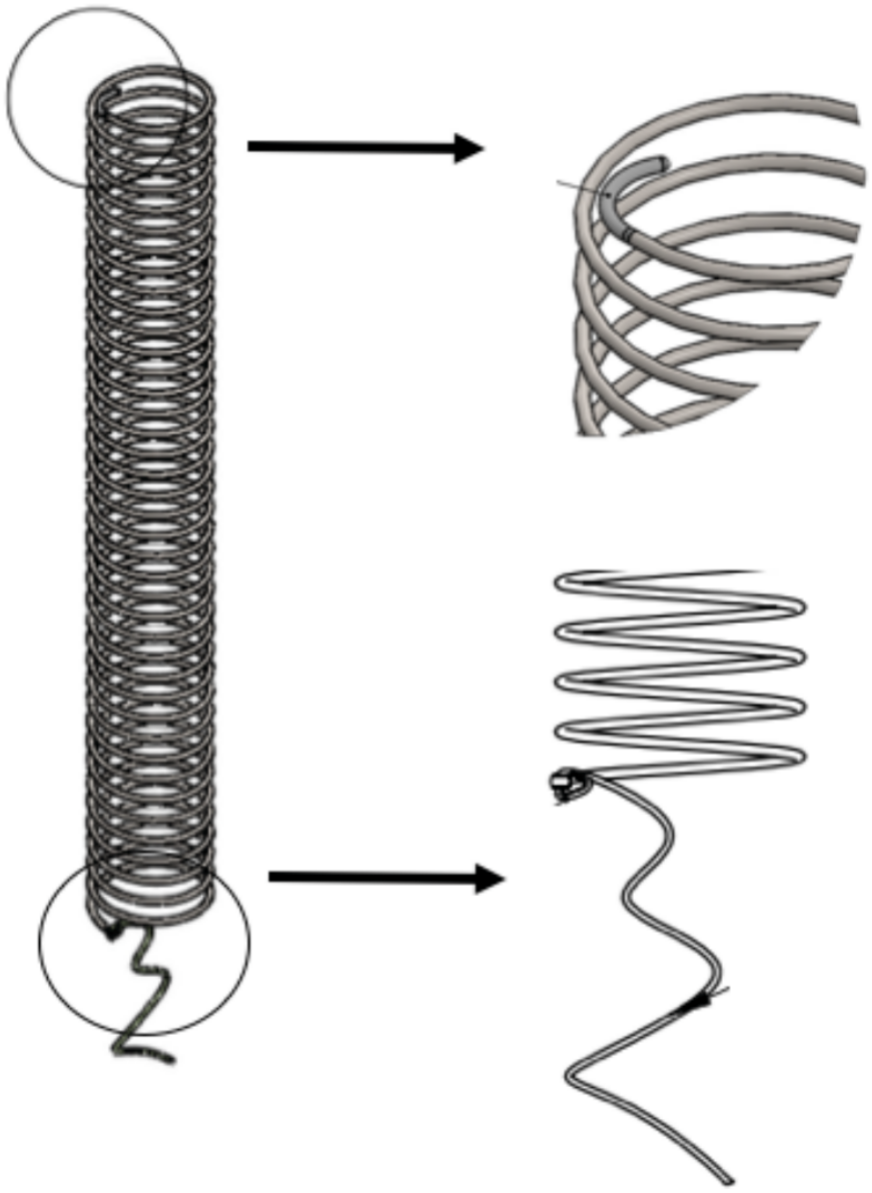
CasMin Twine design. Shape of the twine. Tips characteristics: flexible tip, and tip with suture fixation.

An intra-luminal spiral stent was manufactured for the treatment of tracheomalacia, with an average length of six cm, a 5.5 mm diameter, and a 0.03 mm thickness of stainless-steel. The measures were the same in all animals. The handmade stent for this pilot study is described in the following figure. (Reference number 902711 registered as CasMin-Twine) (Fig. 1).

Before CasMin-Twine implantation, a computerized tomography (CT) scan was performed in one rabbit to measure tracheal diameter and length. Stent size was manufactured 1:1 ratio with tracheal dimensions.

#### Interventional procedure

The rabbits were induced with a combination of medetomidine (0.025 mg/kg intramuscular [IM]) and ketamine (0.15 mg/kg IM), enough to place an intravenous catheter (auricular vein) and introduce a laryngeal mask (V-Gel; Docsinnovent, London, England). Throughout the procedure, the anesthesia was maintained via inhaled isoflurane gas and vital parameters were monitored. It was also used 0.1 ml lidocaine intra-tracheal (Lidocaine 5%) before the delivery of the device.

The device (CasMin-Twine) deployment was performed making a one cm dissection in the ventral region of the neck, at one cm caudal to the cricoid cartilage. At this tracheal level, the intra-tracheal space was punctured via a 21 Gauge needle and administrated 0.1ml of lidocaine 5%. Through the hole made by the needle, the spiral device was introduced with a screwing motion (clockwise) into the tracheal lumen, and the CasMin-Twine proximal tip loop was fixed to the cranial ring with a non-absorbable suture (nylon 6.0 curved needle). The suture is permanent, not only to fix the prosthesis to the tracheal wall but also to locate it in case that is needed to remove the stent. The removal of the stent is possible by cutting the fixation point and unscrewing (counterclockwise) the spiral device.

At the end of the procedure, the position of the device was visualized by fluoroscopy as image study. The stent delivery was always positioned one cm caudal to the cricoid cartilage and one cm cranial to the carina.

### Follow-up

Clinician monthly follow-up was performed with a symptoms checklist to detect any respiratory disorder and other parameters to assess the rabbit’s health. Two experimented veterinarians evaluated the parameters, it included; abnormal appearance (anxiety, depression, inactive or restless), screeches or moans teeth grinding, tonic immobility, rejection of water and / or food and weightless. Cough, dyspnea, inspiratory or expiratory stridor were under respiratory parameters studied and was assigned a number between 0-3, being 0 non-findings and 3 high abnormalities.

### Imaging follow-up

**Figure 2:**
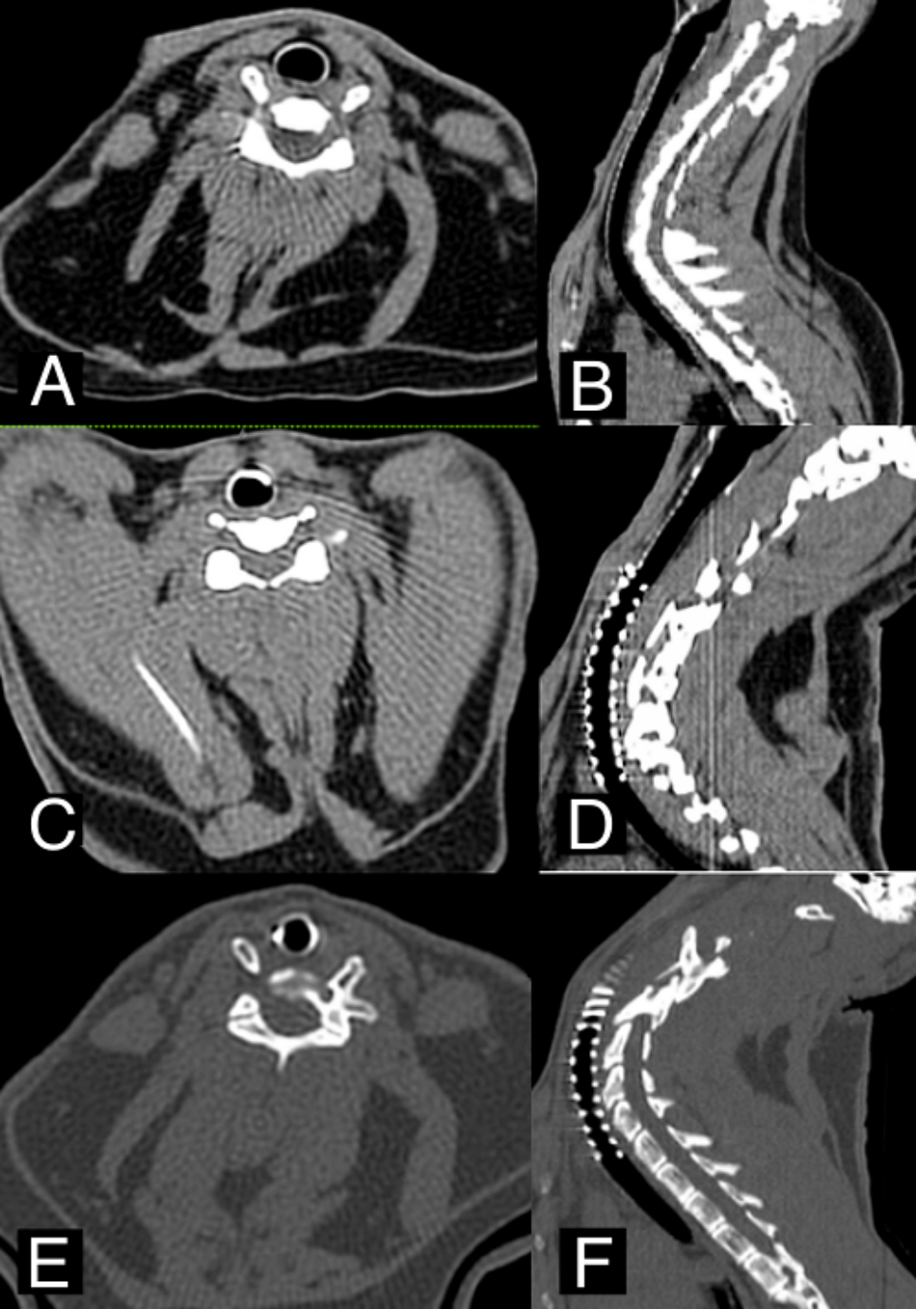
CT images. (A–B) Axial and Sagittal view before CasMin-Twine implantation. (C–D) Axial and Sagittal view 3th month post-implantation. (E–F) Axial and Sagittal view 12th months post-implantation.

A non-contrast-enhanced CT scan was performed after 30, 90 and 365 days (Fig. 2) as an imaging study. Tracheal stenosis, granuloma-presence or tissue overgrowth and mucus accumulation were evaluated. These parameters were previously defined and classified according to previous studies published (Serrano et al., 2016).

Tracheal stenosis was evaluated measuring intra-tracheal lumen, stent thickness and tracheal area: 0 (non-stenosis 0%), 1 (stenosis <20%), 2 (stenosis >20%–<40%), 3 (stenosis >40%–<60%), 4 (stenosis >60%–<80%), 5 (stenosis >80%–<100%).

The granuloma was assessed present or not, and the number of granulomas:

0(non-granuloma),1(presence of one granuloma),2(>1 granuloma).

Mucus accumulation was divided into: 0 (absent), 1 (low accumulation), 2 (moderate accumulation), and 3 (severe retention).

Also, the endoscopic study was performed after 30, 90 and 365 days to evaluate macroscopic findings; inflammatory reaction, granuloma presence, mucous membrane congestion, mucus retentions and endothelization of the prosthesis.

Endoscopy was done with a rigid 30°, four mm optical endoscopy (Karl Storz, Hopkins II).

Degree of endothelization was classified into three groups, visualizing tissue growth over the stent: 0 (no-endothelization: naked stent), 1 (partial endothelization: stent areas not covered), 2 (total endothelization).

Evaluation of mucous membrane congestion was based on erythematous tissue: 0 (non-congestion), 1 (low mucous membrane congestion: orange-colored or patched mucosa), 3 (severe mucous membrane congestion: intense red- bloodshot).

Assessment of the inflammatory reaction was divided into 0: absent, 1: low and 2: mild and 3: severe inflammation.

The amount of mucus retention in tracheal lumen was divided into 0: non-retention, 1: moderate; isolated mucus without air-flow problems, and 2: profuse amounts; ranging from colorless-white appearance of whole stent with difficult airflow (Kilkenny et al., 2010).

Endotracheal granulomas were defined as 0: absent, 1 isolated granuloma and 2 numerous granulomas (Serrano et al., 2016).

### Histopathology: macroscopic and microscopic study

After a period of 365 days, animals were sacrificed by intravenous administration of sodium pentobarbital (120 mg/kg) (Dolethal; Vetoquinol, Lure, France).

The tracheal specimens were removed from the glottis to the carina for its macroscopic evaluation, afterwards, the CasMin Twine was removed and the trachea fixed in 10% formaldehyde. Macroscopic evaluation showed neovascularization and abnormal alterations of the surrounding tissue. Transverse histologic sections were prepared using hematoxylin-eosin stain and were evaluated structural changes of the epithelium and signs compatible with inflammation.

All the data were expressed as mean and SD for qualitative variables. Categorical data were expressed as percentages. A *P* value of <0.05 was considered to indicate statistical significance. Data was processed and analyzed using a computer software program for statistical analyses with SPSS. IBM SPSS Statistics for Macintosh, Version 21.0; IBM Corp., Armonk, NY, USA). Qualitative variables were compared using the Likelihood Ratio test or Fisher’s exact test.

## Results

The ten stented rabbits studied were successfully implanted in 100%. Mean duration of the procedure was 20 min ± 6 min from the anesthetic induction the end of the procedure. The follow-up after the procedure was 12 months, focused on long-term complications.

Behavior and respiratory abnormalities were noted. Two-month post-implantation 23% (3/13) of rabbits presented respiratory stridor during the manipulation, without clinical significance and without relation between imaging findings, and (1/13) one in the control group presented similar symptoms without the association of the presence of tracheal stent.

**Figure 3:**
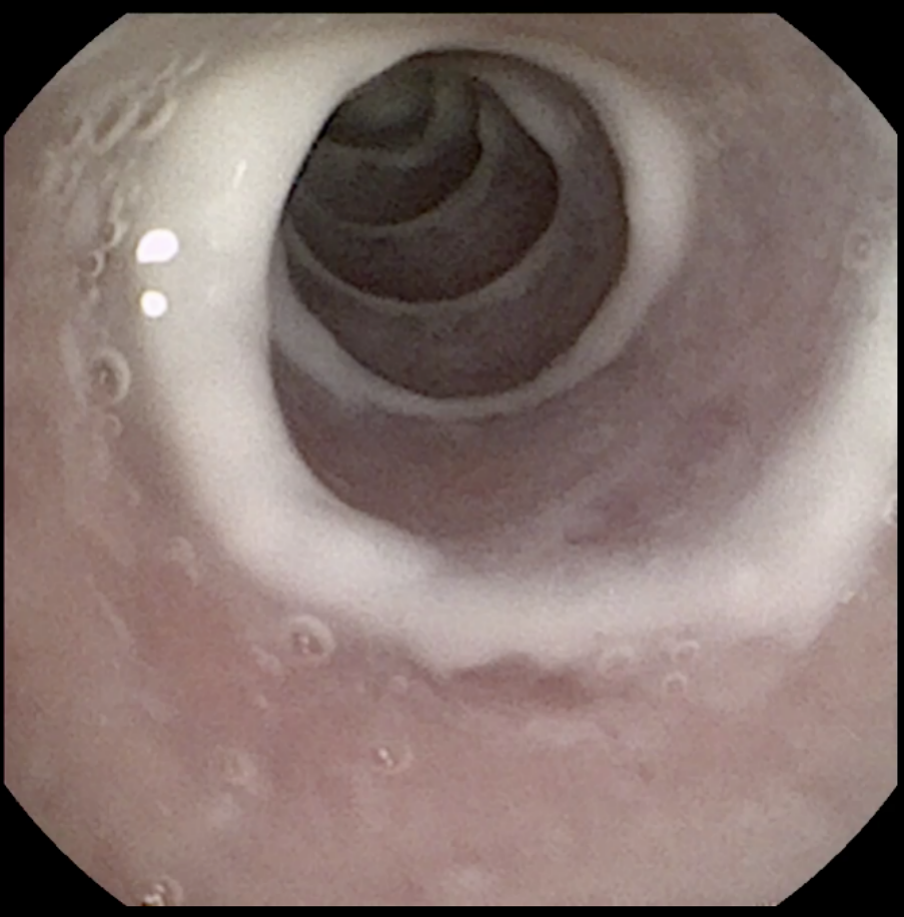
Endoscopic control. 12th month tracheoscopy control: Total endothelization with low mucus retention.

Imaging study results were noted. On first and third month CT study did not show any anatomical change. After 12 months only one animal implanted (10%) showed evidence of granuloma formation in the loop of caudal tip and images compatible with low mucous retention (Fig. 3).

Endoscopic follow-up (Table 1) showed, a partial endothelization categorized as 1 in the 60% of cases at the first control, being total endothelization of the stent after 90 days in 4/6 animals, and in 5/6 after 360 days. Only one animal (10%) still presented areas of stent non-endothelized after one year. No clinical worsening after stent placement was evident in the follow-up period (365 days). Mucous membrane congestion was only visible in 20% (two rabbits) at 90 days and graded as 1 (low congestion).

**Table 1:** Endoscopic follow-up grades. Degree of endothelization: (visualizing tissue growth over the stent) 0 (no-endothelization: naked stent), 1 (partial endothelization: stent areas not covered), 2 (total endothelization). Evaluation of mucosal congestion: 0 (non congestion), 1 (low mucosal congestion: orange colored or patched mucosa), 3 (severe mucosal congestion: intense red- bloodshot). Assessment of the inflammatory reaction 0: absent, 1: low and 2: mild and 3: severe inflammation. Mucus retention in tracheal lumen 0: non-retention, 1: moderate; isolated mucus without air-flow problems, 2: profuse amounts; ranging from colorless-white appearance of whole stent with difficult air-flow. Granulomas 0 absent, 1 isolated granuloma and 2 numerous granulomas. Implanted animals make reference to those animals with CasMin Twine implanted. Control animals were three, they have no endothelization valued because no stent was implanted.

		Implanted animals			Control animals		
		30 days	90 days	365 days	30 days	90 days	365 days
Endothelization	Grade 0						
	Grade 1	60% (6/10)	20% /2/10)	10% (1/10)			
	Grade 2	40% (4/10)	80% (8/10)	90% (9/10)			
Congestion	Grade 0						
	Grade 1	30% (3/10)	30% (3/10)	10% (1/10)		66.6% (2/3)	33.3% (1/3)
	Grade 2						
	Grade 3						
Inflammatory reaction	Grade 0						
	Grade 1	20% (2/10)					33.3% (1/3)
	Grade 2						
	Grade 3						
Mucus retention	Grade 0						
	Grade 1	70% (7/10)	60% (6/10	40% (4/10)			33.3% (1/3)
	Grade 2						
Granuloma	Grade 0						
	Grade 1		10% (1/10)	10% (1/10)			
	Grade 2						

The 60% of the animals presented low mucus accumulation (category 1) at month 12 follow-up, with no significant respiratory abnormalities. Neither mucus accumulation nor mucous membrane congestion showed significant differences with controls (*p* = 1.00 *p* = 0.092).

**Figure 4:**
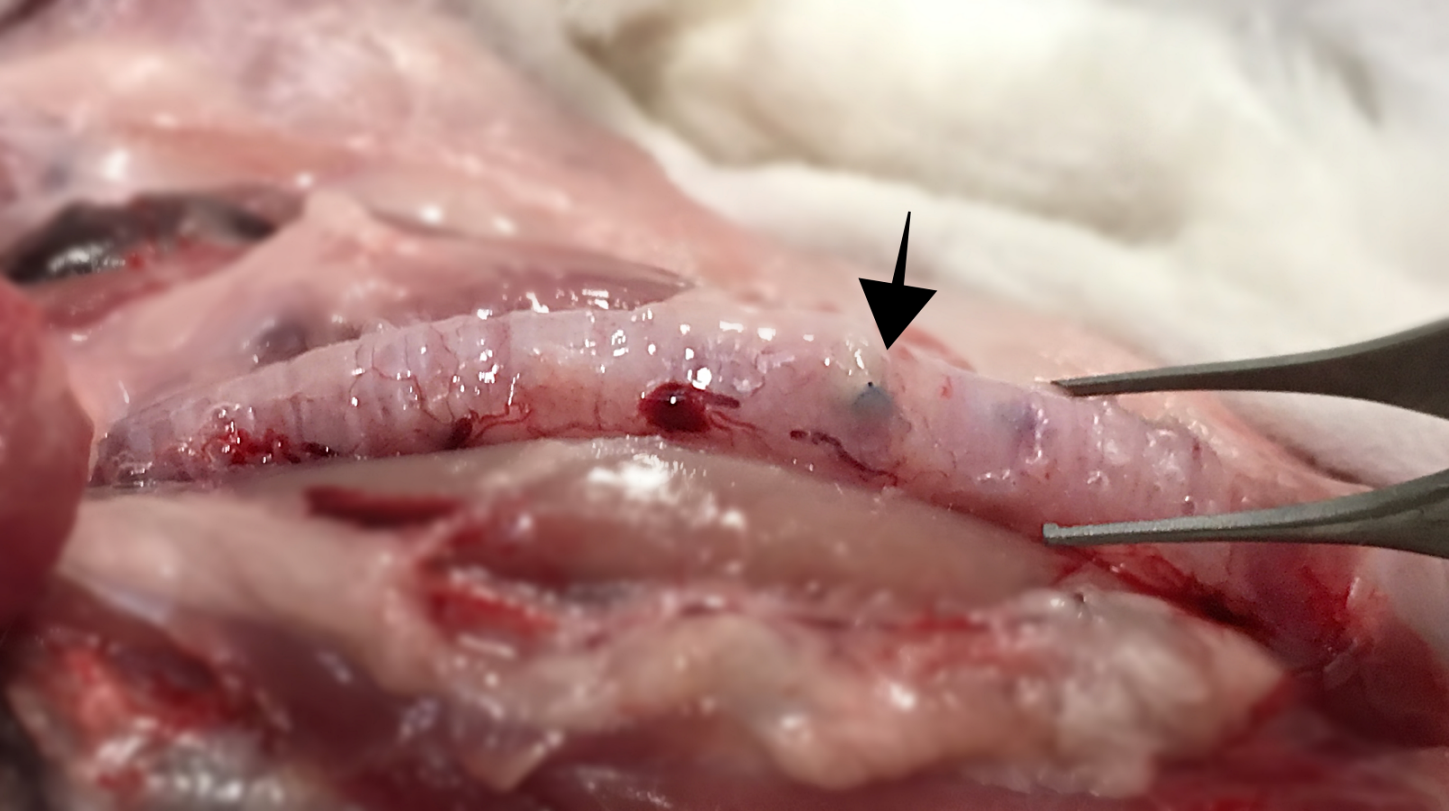
Macroscopic image of an implanted trachea shows no alteration of surrounding tissue. Arrow marks the fixation point of the prosthesis. Photo credit: Jose Rodriguez Gomez.

In the macroscopic evaluation, in one of the implanted animals (1/10) presented neovascularization at the stent fixation point. In 0% of the rabbits the surrounding tissue was altered (Fig. 4).

**Figure 5:**
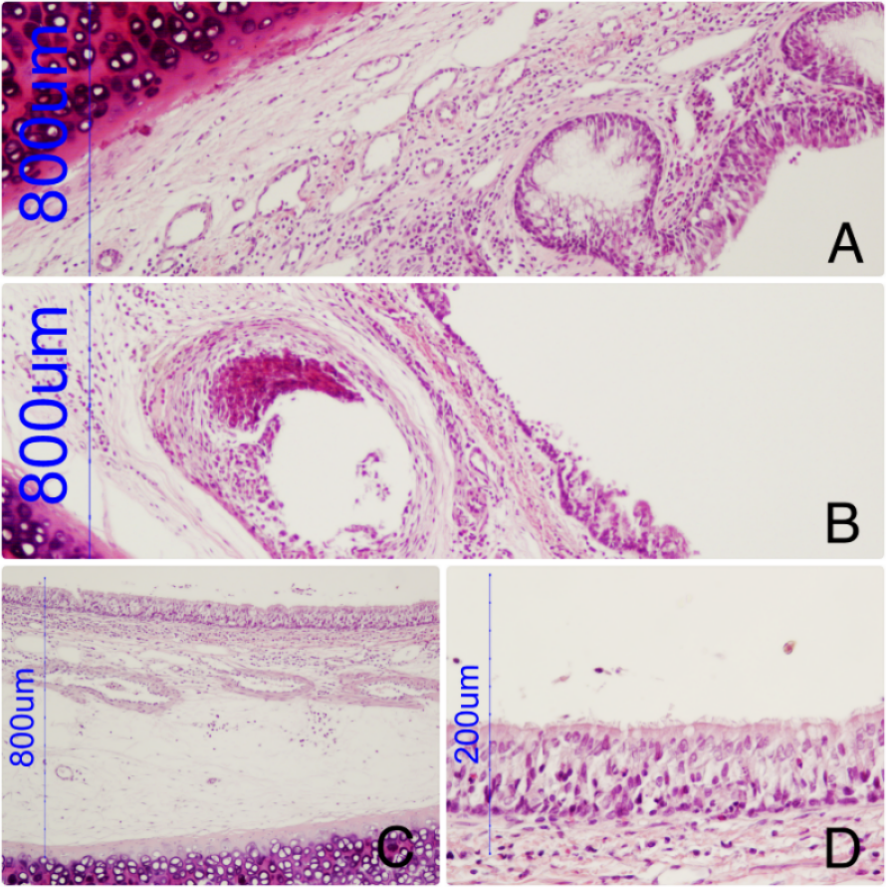
Tracheal wall with a hole corresponding with the device. Discrete inflammatory reaction, slight increase in globet cells. (B) Tracheal wall with a hole corresponding with the device and epithelial absence. (C, D): Prosthesis-free area. Retains normal epithelial morphology and perfect ciliary structure.

The histological study showed mostly discrete inflammatory reaction, slight increase in goblet cells and some areas with loss of ciliary structure. The cartilage structure was not affected. The prosthesis-free areas of trachea maintained the normal histology structure.

The endothelization of the device was confirmed; some areas of the tracheal wall presented holes with a new epithelium surrounding it (Fig. 5).

## Discussion

Tracheal collapse is a pathology which mainly affects middle-aged small and toy dog breeds. High collapse grade can produce serious clinical symptoms with life compromising and negative effects on the patient but also on the owner stress.

Because of the poor results obtained in current treatments (Beranek, Jaresova & Rytz, 2014; Chisnell & Pardo, 2015; Clarke, 2018; Chung et al., 2011; Sun et al., 2008; Herth & Eberhardt, 2016) the search of a new alternative is justified. Medical management is not sufficient and normally is necessary to perform invasive or minimally invasive procedures in uncontrollable patients. These procedures include extra-luminal prosthesis (ELR) and intra-tracheal stents (ITS). They both are available possibilities, but are not exempt from complications. The most appropriate surgical treatment is still controversial. The main goal of tracheal collapse procedures is to give permanent support and retrieve the normal anatomy without changing the luminal ciliary structure.

Placement of ELR requires invasive tracheal dissection and high morbidity has been associated with its use for intra-thoracic collapse, for this reason now is contraindicated in this localization (Chisnell & Pardo, 2015). Chisnell & Pardo (2015), studied 23 dogs treated with ELR, complications arising tracheal dissection were 1/23 death during the procedure and a 17% presented laryngeal paralysis.

A previous report (Tinga et al., 2015) of 73 dogs treated with ELR described a 42% of major complications; short-term complications include a 26% death related to respiratory compromise and 15% of laryngeal paralysis. In this study, long-term follow-up was performed in 40 patients, with 15% of deaths associated with respiratory abnormalities and 3% of laryngeal paralysis. Patients under ELR treatment were mostly cervical collapse (60%), reported deaths were associated with the progression of this pathology and the collapse of the entire trachea.

Similar laryngeal paralysis damage and deaths related to respiratory abnormalities have been also reported (Becker et al., 2012). In this study, showed no survival difference between dogs with simple cervical collapse with those with entire tracheal affectation. However, other authors have reported significant differences (Chisnell & Pardo, 2015; Tinga et al., 2015; Sun et al., 2008).

Even though our procedure requires some tissue dissection, there were no problems associated with tracheal exposure. The CasMin-Twine procedure only requires one cm of ventral dissection to expose cartilage rings, whereas the ELR needs 360° dissection affecting surrounding tracheal structures and an experimented surgeon is required. Moreover, total support of entire trachea is currently recommended (Clarke, 2018; Raske et al., 2018) and with the CasMin-Twine procedures, it is feasible to give support to both extra and intra-thoracic trachea with a single spiral stent without having complications compared to ELR.

An intra luminal prosthesis or stent can be manufactured with different materials, silicone stents and metallic stents (MS) (Saito & Imamura, 2005; Folch & Keyes, 1998). In human patients, the Food and Drug Administration (FDA) prohibited every kind of metallic stents for benign airway disease due to the major complications and no recovery possibility of the devices (Chung et al., 2011; Saito & Imamura, 2005; Folch & Keyes, 1998; Onofre et al., 2008). Even though this warning, self-expanding mesh-shaped nitinol stents (SEN) are the best available option for canine tracheal collapse (Weisse et al., 2008; Serrano et al., 2016; Raske et al., 2018; Weisse et al., 2019). Silicon stents are not recommended in veterinary practice due to the high migration and excessive mucus accumulation already described (Onofre et al., 2008).

There is a small number of published studies which describe the stenting procedure in canine tracheal collapse, and most of them reported similar complications: granuloma formation, stent migration, severe mucus accumulation and stent fracture (Rosenheck et al., 2017; Serrano et al., 2016; Moritz, Schneider & Bauer, 2004; Raske et al., 2018; Weisse et al., 2019; Saito & Imamura, 2005). A critical key point in this procedure is to choose precise stent sizing and handicraft/surgical skills (Clarke, 2018).

The current recommendation is to overestimate stent diameter between 10–20% to reduce migration, the measurement of tracheal diameter is essential, but also a complex procedure (Sura & Krahwinkel, 2008; Rosenheck et al., 2017; Raske et al., 2018; Johnson, Singh & Pollard, 2015). In our study with CasMin-Twine, it is not necessary to overestimate the diameter of the device because the prosthesis is fixed by suture knot and not by radial force. We measured the tracheal diameter with CT and the same size for stent was implanted. No migrations were present in controls; the fixing suture maintains the CasMin-Twine device in position and this allowed not to have perfect precision in the diameter size measurement.

In a study performed by Moritz, Schneider & Bauer (2004), 24 dogs underwent endotracheal biliary Wallstent (auto-expandable stainless-steel stents) with an early improvement of clinical signs and two deaths in the short-term. five patients of eighteen (27.77%) developed granulomas and 83.3% of cases showed a shortening of the stent. Currently, nitinol stents are recommended (Weisse et al., 2008; Clarke, 2018; Serrano et al., 2016; Sun et al., 2008; Weisse et al., 2019); however, it presents similar results regarding complication compared with MS, and also are not removable after endothelization (Saito & Imamura, 2005). In our pilot, we obtained highly acceptable results with stainless steel prosthesis, achieving better results than previous experiences in rabbit model (Serrano et al., 2016). This device was retrievable even though endothelization without complications, no-bleeding or tissue destruction occurs due to the new surgical approach.

## Conclusion

The technical success in this study was optimal; it demonstrates the efficacy and low risk arising from the surgery approach.

Tracheal tissue reactivity to the CasMin-Twine was minimized, and spiral shape allowed to remove the stent without tissue damage.

Neither ITS nor ELR associated complications occurred in this study.

The limitations of this study were a small sample size as it was a pilot study with only ten healthy animals, and finally its results could not be extrapolated directly to other animal species or humans.

The satisfactory results obtained in comparrison with tracheal stent studies in the same animal species (Serrano et al., 2016), give to veterinarians a new option for this complicated pathology. Clinical trials and expanded analysis will be needed.

##  Supplemental Information

10.7717/peerj.7797/supp-1 Data S1 Raw data

## Additional Information and Declarations

### Data Availability

The following information was supplied regarding data availability: Raw data is available as a Supplemental File.
